# PGC-1α in Disease: Recent Renal Insights into a Versatile Metabolic Regulator

**DOI:** 10.3390/cells9102234

**Published:** 2020-10-03

**Authors:** Joseph M. Chambers, Rebecca A. Wingert

**Affiliations:** 1 College of Pharmacy, Natural and Health Sciences, Manchester University, Fort Wayne, IN 46845, USA; 2 Department of Biological Sciences, Center for Stem Cells and Regenerative Medicine, Center for Zebrafish Research, Boler-Parseghian Center for Rare and Neglected Diseases, University of Notre Dame, Notre Dame, IN 46556, USA

**Keywords:** PGC-1α, disease, kidney, cancer, AKI, CKD, nephron, PKD, cilia, cystogenesis

## Abstract

Peroxisome proliferator-activated receptor gamma co-activator 1 alpha (PGC-1α) is perhaps best known as a master regulator of mitochondrial biogenesis and function. However, by virtue of its interactions as a coactivator for numerous nuclear receptors and transcription factors, PGC-1α also regulates many tissue-specific tasks that include adipogenesis, angiogenesis, gluconeogenesis, heme biosynthesis, thermogenesis, and cellular protection against degeneration. Knowledge about these functions continue to be discovered with ongoing research. Unsurprisingly, alterations in PGC-1α expression lead to a range of deleterious outcomes. In this review, we provide a brief background on the PGC-1 family with an overview of PGC-1α’s roles as an adaptive link to meet cellular needs and its pathological consequences in several organ contexts. Among the latter, kidney health is especially reliant on PGC-1α. Thus, we discuss here at length how changes in PGC-1α function impact the states of renal cancer, acute kidney injury (AKI) and chronic kidney disease (CKD), as well as emerging data that illuminate pivotal roles for PGC-1α during renal development. We survey a new intriguing association of PGC-1α function with ciliogenesis and polycystic kidney disease (PKD), where recent animal studies revealed that embryonic renal cyst formation can occur in the context of PGC-1α deficiency. Finally, we explore future prospects for PGC-1α research and therapeutic implications for this multifaceted coactivator.

## 1. Introduction

Peroxisome proliferator-activated receptor gamma co-activator 1 alpha (PGC-1α, or alternatively PPARGC1A) serves as a master regulator for mitochondrial biogenesis and function. Mitochondria are classically defined as the powerhouse of the cell, as they function to produce the essential energy needed for cellular processes. PGC-1α was first discovered in the late 1990s as its expression was induced in brown adipose tissue during the response to cold temperature exposure [1]. PGC-1α was found to alter the transcriptional activities of several key mitochondrial genes, which ultimately resulted in increased mitochondrial DNA [1]. Due to its physical interaction with the nuclear hormone receptor peroxisome proliferator-activated receptor gamma (PPARγ) that led to an increase in PPARγ transcriptional activity, this novel co-activator acquired its name, PGC-1α [1]. Interestingly, PGC-1α was shown in the same study to also bind a number of other nuclear hormone receptors including thyroid hormone receptor (THR), retinoid X receptor α (RXRα), and estrogen receptor (ER) [1]. Soon after, the glucocorticoid receptor (GR) was identified as another nuclear receptor binding partner [2]. This was just the beginning of research to uncover the multitude of critical roles that PGC-1α and its family members play during the molecular workings of cellular life [3,4,5,6,7].

It is now appreciated that the PGC-1 family of transcriptional coactivators hold key positions in controlling many cellular pathways, among these figuring prominently in regulating metabolism as well as conducting a host of tissue-specific functions [3,4,5,6,7]. These coactivator proteins do not bind DNA directly but rather all work through interactions with transcription factors and other proteins to influence gene expression. The family consists of three related members: PGC-1α, PGC-1β, and PGC-related coactivator (PRC) [1,8,9]. The PGC-1 family members are conserved across higher vertebrates, including fish, amphibians, birds, and mammals [3,4,5,6,7]. Thought initially to be absent from the genomes of lower eukaryotes such as yeast, insects and worms [2,3], a PGC-1 homologue named Spargel was later identified in *Drosophila*, where it similarly functions in mitochondrial metabolism and regulates insulin signaling [10].

PGC-1 family members share a related overall structure, with functional regions that include an N-terminal activation domain, a central regulatory domain, and a C-terminal RNA recognition motif [2,3,4,5,6]. The activation domain can bind several proteins that possess histone acetyltransferase (HAT) activity and influence chromatin structure [3,4,5,6,7]. The central domain is known to dock various transcription factor targets, but the latter are not limited to interacting with only this region [3,4,5,6,7]. The RNA binding domain enables interactions with proteins of the mediator complex that interacts with the RNA polymerase II machinery [3,4,5,6,7]. Among the family members, PGC-1β shows the closest homology to PGC-1α, and both of these proteins are abundant in tissues with high oxidative metabolism [3,4,5,6,7]. PGC-1α and PGC-1β are the best studied members of this family, and in line with their expression pattern, they both participate in regulating mitochondrial functions [3,4,5,6,7]. In contrast, PRC is expressed nearly ubiquitously [9] but its roles remain comparatively understudied [6,7].

To date, the PGC-1 coactivators have been catalogued to interact with over 20 different transcription factors in sum, with some shared targets and others unique to just one family member [3,4,5,6,7]. These targets are not limited to nuclear receptor family members (such as the previously mentioned PPARγ, THR, ER, RXRα, and GR) but include binding to unliganded nuclear receptors, forkhead/winged helix proteins, zinc-finger proteins, and others [3,4,5,6,7]. Despite large-scale efforts to identify partners such as through genome-wide coactivation screens [10], it is likely that ongoing research will only continue to reveal additional binding partners. Interestingly, the target binding activities of PGC-1α are influenced by numerous post-translational modifications that include acetylation, methylation, phosphorylation and SUMOylation, although comparatively less is known about the influence of such modifications on the other family members [3,4,5,6,7]. Additionally, as we will discuss later in the present work, both PGC-1α and PGC-1β are alternatively spliced, but the functional consequences of this are not yet fully appreciated [3,4,5,6,7].

Today, PGC-1α is known to be specifically expressed in organs with high energy demands such as the heart, kidney, brain, and skeletal muscles, among others [3,4,5,6,7]. Across all of these organs, expression of PGC-1α is highly regulated by a number of signal transduction pathways and hormones to maintain metabolic balance in a tissue specific manner [3,4,5,6,7]. Furthermore, in such locations as brown fat and the liver, dynamic changes in PGC-1α expression are used to respond to fluctuating physiological and environmental stimuli [11]. These changes can consist of cold exposure, exercise, fasting, etc. In the following paragraphs, we provide an overview of some example signaling mechanisms that regulate PGC-1α expression in response to particular stimuli to illustrate the diversity of ways in which PGC-1α can be employed to regulate biological processes.

Cold-induced stress and the cellular response, commonly referred to as adaptive thermogenesis, is essential to maintain body temperature thus ensuring proper organ function. Interestingly, upon subjecting PGC-1α null mice to a standard cold challenge, the animals were unable to maintain core body temperatures and succumbed to hypothermia when exposure was extended beyond 6 h [12]. Defense of body temperature is a functional readout of brown adipose tissue; therefore, this study was one of the first indications that PGC-1α had a molecular role in this cell type [12]. In both brown fat and skeletal muscle, PGC-1α expression is induced by cold and regulates a suite of nuclear and mitochondrial-encoded genes corresponding to subunits of the electron transport chain [7,12]. For example, in brown fat, PGC-1α increases transcriptional activity at the promoter of mitochondrial uncoupling protein-1 (UCP1), where subsequent UCP1 activity results in the production of heat by dissipating the mitochondrial proton gradient [13,14,15,16].

Fasting induces PGC-1α expression in the liver through the activation of p38 mitogen-activated protein kinase (p38), where PGC-1α interacts with transcription factors to enable the body to adapt to nutrient deprivation [3,17,18]. PGC-1α is sufficient to activate the processes of gluconeogenesis, fatty acid B-oxidation, ketogenesis, heme biosynthesis, and bile-acid homeostasis. Each of these processes is triggered by PGC-1α coactivation of specific hepatic transcription factors [17,18,19,20,21]. Without PGC-1α, fasted mice develop hypoglycemia and hepatic steatosis [22]. Further, it was recently demonstrated that PGC-1α plays an important role in the hepatic response to insulin during the fasting-to-fed transition by regulating expression of upstream components of the insulin-signaling pathway [23,24].

PGC-1α after exercise-induced cellular stress has been extensively studied in skeletal muscle. In this context, several pathways previously discussed remain relevant, with the overall goal of equilibrating cellular metabolism. In this situation and tissue type, investigators have found several modes of action that result in a PGC-1α-dependent adaptive response. First, nerve stimulation activates the calcium/calmodulin-dependent protein kinase IV (CaMKIV) and calcineurin A (CaN) pathway and increases PGC-1α expression via the myocyte enhancer factor 2C and 2D (MEF2C/2D) [15,19] or by way of the CaMKIV target cAMP response element binding protein (CREB) [15,25,26,27,28,29,30]. Another previously mentioned pathway involved in both fasting-induced PGC-1α expression and exercise-induced PGC-1α expression is p38 MAPK signaling. Interestingly, multiple factors can be initiated by p38 MAPK that result in downstream PGC-1α functions. Both MEF2 [30] and ATF2 [31] can increase expression of PGC-1α in muscle post exercise.

Much of this information was gathered in the years immediately following the discovery of PGC-1α and laid the groundwork for subsequent studies. This next wave of studies brought to light the extensive network of PGC-1α functionality and its diverse impacts on physiology during health and disease states. Further, recent studies have begun to highlight important developmental roles for PGC-1α. In Section 2 of this review, we will discuss several cellular and molecular roles of PGC-1α during health and disease progression. In Section 3 of this review, we explore the functions of PGC-1α in renal disease and development.

## 2. PGC-1α in the Context of Human Disease and Development

**Figure 1:**
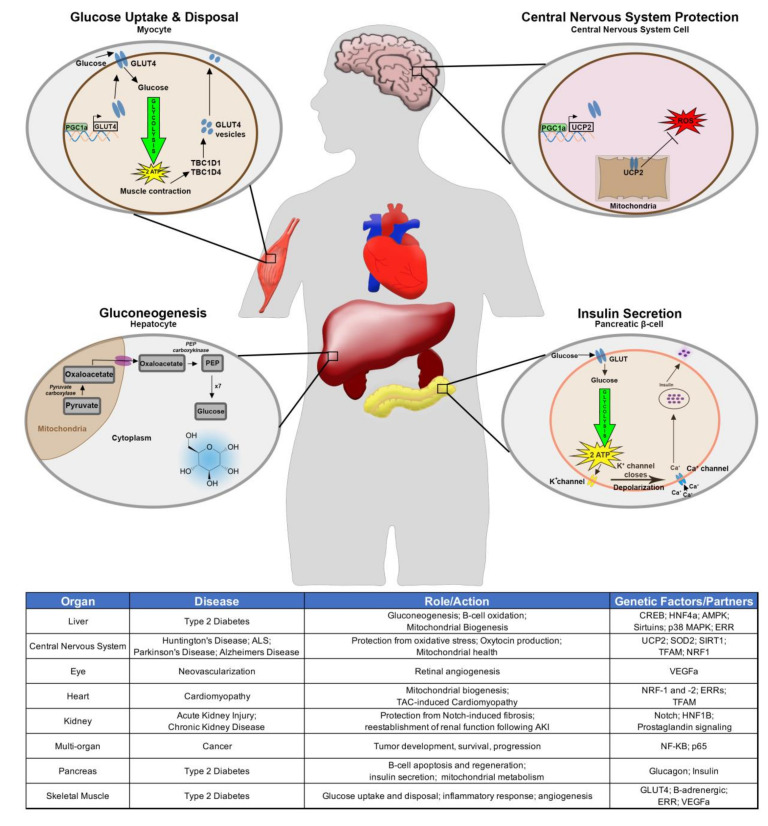
Systems affected and corresponding disease states in peroxisome proliferator-activated receptor gamma co-activator 1 alpha (PGC-1α) contexts. Schematic depicting the role of PGC-1α in select cellular processes including glucose uptake and disposal via glucose transporter 4 (GLUT4) in skeletal muscle (top, left), central nervous system reactive oxygen species (ROS) protection via PGC-1α uncoupling protein-2 (UCP2) (top, right), gluconeogenesis in the human liver (bottom, left), and insulin secretion from a pancreatic B-cell (bottom, right). Table summarizes key disease states and the associated organ systems in addition to the role PGC-1α plays in those organs and some of the genetic interactions with PGC-1α.

There has been extensive research regarding the impact of PGC-1α function in human disease (Figure 1). More recently, there is a growing body of work that has begun to reveal insights regarding the roles of PGC-1α in developing tissues. The foundational information presented above illustrates the functions of PGC-1α in the adaptive response to several stimuli including exercise and fasting. Not coincidentally, the relationship between inactivity and poor diet can lead to type 2 diabetes. Type 2 diabetes mellitus is a metabolic disorder caused by abnormal glucose metabolism due to insulin resistance or disrupted B-cell function culminating in abnormal insulin production.

Here, we will highlight part of the relationship between PGC-1α and tissues in terms of type 2 diabetes mellitus. A comprehensive description of these relationships is nicely detailed in another review [30]. As previously discussed, in the liver PGC-1α acts to 1) maintain proper levels of gluconeogenesis with well-studied pathways including CREB, HNF4a, AMPK, Sirtuins, and p38 MAPK 2) control B-cell oxidation and 3) regulate mitochondrial biogenesis by partnering with estrogen related receptors (ERRs) and other factors [17,18,19,20,21,22,30]. Understandably PGC-1α is also linked with type 2 diabetes mellitus in the pancreas where B-cells are supposed to manufacture and secret insulin. Early studies found PGC-1α overexpression can decrease B-cell insulin secretion [31]. Further research has found roles for PGC-1α in B-cell apoptosis, regeneration, insulin secretion, and mitochondrial metabolism [32,33,34,35]. While much of the attention on type 2 diabetes is understandably focused on the liver and pancreas, another largely affected system are skeletal muscles that rely on glucose energetics. PGC-1α activates the glucose transporter 4 (GLUT4) and in doing so can stimulate glucose uptake. This relationship between PGC-1α and glucose in skeletal muscles is extended to the process of glucose disposal [30,36].

A number of additional studies have demonstrated the importance of PGC-1α in skeletal muscle, perhaps interrelated to type 2 diabetes mellitus. Interestingly, researchers discovered a molecular node connecting the metabolic factors PGC-1α/PGC-1β with immune pathways, specifically the inflammatory response of NF-kB. For example, PGC-1α/PGC-1β repress NF-kB by reducing phosphorylation of p65 in skeletal muscle [37]. Another important association that could impact the relationship between PGC-1α and type 2 diabetes is the discovery of key molecular events connecting exercise to PGC-1α driven angiogenesis in skeletal muscle. This important tie is corroborated by phenotypes observed in PGC-1α deficient mice. As compared to wild-type counterparts, PGC-1α-null mice did not undergo robust angiogenesis post exercise [38].

Further investigation revealed a deeper mechanistic insight, which placed B-adrenergic signaling upstream of PGC-1α in this process and also relies on ERR to activate VEGF signaling to induce angiogenesis [38]. The role PGC-1α plays in angiogenesis is not limited to skeletal muscle as Saint-Geniez et al. (2013) provide evidence PGC-1α controls retinal angiogenesis by inducing VEGFa in multiple retinal cell types, including Muller cells, ganglion cells, photoreceptors, and retinal pigment epithelial cells [39]. Additionally, PGC-1α is highly upregulated in the retina during postnatal development in mice, especially from P5-P17 [39]. Consequently, mice deficient in PGC-1α have signs of reduced retinal angiogenesis in both early developmental stages and adulthood [39]. Further investigation revealed that PGC-1α is involved in pathological neovascularization [39].

A principal area of study has identified roles for PGC-1α in heart development and maturation. A number of compelling findings have been summarized previously [40]. To highlight some of these findings, Lai et al. (2008) show intriguing data indicating a level of redundancy in mice when it comes to the roles of family members PGC-1α and PGC-1b [41]. Mice deficient in one factor develop relatively normally; however, when both factors are knocked out, the compound mutant mice develop a number of heart defects and perish shortly after birth [41]. These redundant layers of regulation are also observed in murine brown adipose tissue [41]. Other studies have suggested timing of the knockout may affect heart function as well [42,43,44,45]. As expected from the contents discussed thus far, PGC-1α is responsible for mitochondrial biogenesis in the heart through its relationships with genetic partners, including TFAM, NRF-1, NRF-2, and ERRs [40,43,45,46]. Additionally, loss of PGC-1α results in cardiomyopathy following transverse aortic constriction [44]. Importantly, PGC-1α expression is able to overcome cardiomyopathy, although this depends on the time of induction [43]. Findings indicating the significance of timing and redundancy should elicit a response in the research community to identify the possibility of this relationship in other developmental contexts.

PGC-1α is important for removing reactive oxygen species (ROS). This is especially important for neurological diseases where PGC-1α has been shown to decrease ROS and protect neural cells from oxidative stress by inducing expression of several detoxifying factors such as UCP2 and SOD2 [47]. The link between PGC-1α and neurobiology involves developmental aspects and disease contexts including Alzheimer’s disease, Huntington disease, and amyotrophic lateral sclerosis (ALS), among others. In a developmental context, Blechman et al. (2011) found a direct correlation between PGC-1α and oxytocin production in the hypothalamus [48]. The well-studied SIRT1/PGC-1α relationship is very important for mitochondrial recovery following intracerebral hemorrhage in rats [49]. In the case of Huntington disease, PGC-1α and its downstream partners TFAM and NRF-1 are needed to prevent mitochondrial dysfunction that leads to the disease progression [50]. An important finding to both the neuroscience community as well as those interested specifically in PGC-1α came in a 2012 publication that identified several novel PGC-1α isoforms that dictate age of onset for Huntington’s disease [51]. These and other tissue and function-specific isoforms are nuances of studying PGC-1α [52]. On a cellular level, PGC-1α impacts ribosomal transcription, which has recently been found to impact Huntington disease progression [53]. Similarly, the development of Parkinson’s disease entails central nervous system specific PGC-1α isoforms [54,55]. This same approach is a focus of Alzheimer’s disease, where PGC-1α can reduce β-secretase thereby decreasing amyloid-β and the associated neuronal and cognitive loss [56]. A comprehensive understanding of PGC-1α and Alzheimer’s disease has been previously reviewed [57].

As cancer studies have shifted to understanding the metabolism of tumors and the tumor microenvironment, there have been several studies focused on how PGC-1α might affect tumorigenesis and metastasis. Currently there is not consensus in the literature regarding PGC-1α levels and the impact on tumorigenesis. Different types of cancers exhibit different PGC-1α expression trends. Further, some cancers such as breast, colon, ovarian, and melanomas have observed both increased and decreased expression of the gene in tumor tissue. The conclusions reached in a very informative review by Mastropasqua et al. (2018) indicate PGC-1α responds to cellular needs in terms of energy demands [58]. This conditional complexity can be mostly explained by the dynamic nature of the PGC-1α gene in combination with the complicated mechanisms leading to cancer progression; therefore, the interface of these two issues presents challenges to conducting research in this area [58]. In addition, PGC-1α has many upstream and perhaps an even greater number of downstream effectors that allow for adaptive responses thus making a simple linear relationship with cancer highly unlikely. An accurate understanding of the role of PGC-1α in cancer will most likely be dependent on the molecular subtype and tissue of origin. The role of PGC-1α cellular metabolism, specifically of cancer cells, in addition to budding therapeutic options, are nicely described in another review by Bost and Kaminski [59]. We will further discuss kidney cancer below.

## 3. PGC-1α in Kidney Disease and Development

The kidney requires an abundance of energy because of the essential roles it carries out as a vital organ. These functions include blood filtration, ion transport, fluid homeostasis, and waste removal. The nephron is the functional unit of the kidney and is composed of three main components: (1) the glomerulus functions to filter the blood, (2) the segmented tubule that modifies the filtrate, and (3) the collecting duct system that transports waste for excretion [60]. Genetic analyses have illuminated that PGC-1α is necessary for proper nephrogenesis in the zebrafish [61,62]. However, other vertebrate species with PGC-1α loss of function models have not presented with explicit kidney developmental phenotypes in PGC-1α deficient contexts [63]. Interestingly, there is precedence in mice that PGC-1α deficiency can be masked by PGC-1β compensation [41]. Perhaps future studies will shed light on potential for redundant or partially overlapping functions for PGC-1α and PGC-1β in the kidney.

**Figure 2:**
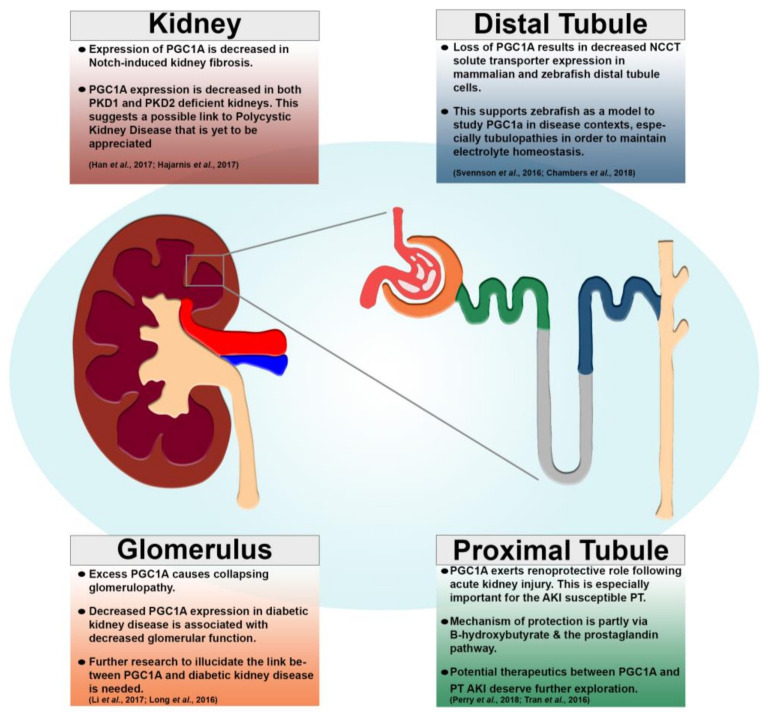
Kidney disease states associated with PGC-1α expression. Renal diagram illustrating general (top, left) and nephron compartment-specific functions of PGC-1α in addition to areas of future research. Kidney (left, maroon), nephron (right), glomerulus (orange), proximal tubule (green), and distal tubule (dark blue). References for cited studies listed within. (PT = proximal tubule, AKI = acute kidney injury).

Nevertheless, as one of the most energy demanding organs, it is logical that PGC-1α is highly expressed in the kidney. This expression is conserved across vertebrate species spanning zebrafish, mice, and humans [61,63,64,65,66,67]. Though studies to date have not identified overt loss of function renal phenotypes in mammalian development, there exists a vast amount of literature suggesting a link between PGC-1α and reno-protective properties at later stages. Subjects deficient in PGC-1α experience deleterious outcomes after suffering from acute kidney injury (AKI) as they progressively undergo renal fibrosis and suffer from chronic kidney disease (CKD). In the following subsections, we will detail a number of these studies and key findings, as there is a growing body of evidence revealing critical roles for PGC-1α and regulation of mitochondria biogenesis in kidney development and disease (Figure 2).

### 3.1. Kidney Cancer

When basal expression of PGC-1α is disrupted in the kidney, the risk of cancer increases. As previously discussed, the exact role of PGC-1α in cancer is not entirely understood, where research has revealed that PGC-1α can be is upregulated or downregulated in a multitude of cancer types [58]. In the context of kidney cancer, the lack of a clear correlation between PGC-1α expression and cancer development and progression is unfortunately only further perplexing. For example, in the context of Birt–Hogg–Dube (BHD) syndrome both PGC-1α and the tumor suppressor FLCN are altered resulting in predisposition to patients developing lung cysts, hair follicle tumors, and renal cancer [68]. FLCN loss induces a metabolic shift toward oxidative phosphorylation and elevated mitochondrial biogenesis in a PGC-1α-dependent manner [68]. Inactivation of PGC-1α in cancer cells rescued hyperplastic phenotypes in FLCN-null kidneys [68]. Together this data indicates that increased FLCN deficiency and subsequent upregulation of PGC-1α enhances energy production and provides malignant tumor cells a growth advantage that fuels renal carcinogenesis [68].

Contrary to BHD syndrome, the development of clear cell renal carcinoma (ccRCC) involves the downregulation of PGC-1α by a HIF/Dec1-dependent mechanism. The most common genetic driver of ccRCC is the tumor suppressor Von Hippel–Lindau (Vhl), which, when mutated, promotes constitutive expression of HIF-a and promotes metastasis, invasion, angiogenesis, and metabolism [60]. PGC-1α expression in VHL-deficient ccRCC cells restores mitochondrial function, upregulated antioxidant gene expression, suppresses tumor growth, and sensitizes cancer cells to cytotoxic chemotherapy and radiotherapy treatments [69]. ERRα was demonstrated to be an essential co-regulator of PGC-1α in mediating mitochondrial biogenesis in the context of ccRCC [69]. ccRCC is considered a metabolic disease that manifests histological hallmarks such as lipid accumulation and lipid storage, which is thought to support tumor survival. The Cancer Genome Atlas and other data repositories indicate PGC-1α expression is significantly decreased in ccRCC tissues versus healthy kidneys and is associated with poor patient prognosis. A study found supplying ccRCC cells with melatonin restores PGC-1α levels, thereby eliminating abnormal lipid accumulations and inhibiting tumor progression [70]. Furthermore, this melatonin-initiated rescue was driven by a novel ‘tumor slimming’ mechanism where lipid droplets are broken down via PGC-1α/UCP1-mediated autophagy and lipid browning [70]. Another research group described a novel role for PGC-1α, whereby the PGC-1α/miR-29 axis attenuates the epithelial-to-mesenchymal transition (EMT) program to prevent ccRCC tumor progression [71]. Restoration of PGC-1α in ccRCC cells suppresses collagens associated with invasive and migratory behaviors via activation of miR-29a [71]. PGC-1α-mediated repression of collagen/DDR1 signaling culminates in downregulation of known EMT genes SNAIL1 and SNAIL2, which curbed pro-invasive phenotypes [71].

A genetic loss of function screen revealed MYBBP1A promotes tumorigenesis in absence of glucose by operating a “metabolic switch” involving PGC-1α [72]. In multiple ccRCC lines, downregulation of MYBBP1A induces tumor cell survival and proliferation [72]. In response to decreased MYBBP1A, c-MYB repression is alleviated leading to transcriptional activation of PGC-1α, which elicits a shift to OXPHOS metabolism [72]. This data indicates PGC-1α is a stress sensor of glucose depletion and can provide tumor cells a competitive advantage in restrictive microenvironments similar to previously discussed roles for PGC-1α above [72]. Evaluation of biopsies from 380 ccRCC patients revealed increased MLXIPL and decreased PGC-1α mRNA levels in the tumor microenvironment are significantly correlated with poor overall survival [73]. Further, elevated MIXIPL and PGC-1α were associated with a decline in the number of B cells, CD8+ T cells, macrophages, neutrophils, and dendritic cells [73]. Thus, MIXIPL and PGC-1α signatures are closely related to the degree of immune infiltration, underscoring the potential of monitoring and targeting the tumor microenvironment in ccRCC [73].

### 3.2. Acute Kidney Injury (AKI)

Acute kidney injury (AKI) is the sudden and rapid decrease in kidney function resulting from a severe insult [74]. This insult can originate from various sources, such as decreased blood flow (ischemia), increased inflammation, introduction of a toxin perhaps in the form of a chemical or antibiotic, or obstructed urine flow. These insults can cause damage to cells throughout the nephron functional units and/or to the interstitium [74]. AKI can be detected clinically by evaluating a number of kidney related outputs including blood urea nitrogen, creatinine, and atypical ion levels.

Over the last decade, a better understanding of the cellular energy defects associated with various types of AKI has understandably focused on the role PGC-1α plays during and after AKI. One of the first studies that examined the specific relationship between AKI and PGC-1α was published over a decade ago. Rasbach and Schnellmann hypothesized that increasing mitochondrial biogenesis could help overcome ischemic injury conditions [75]. They discovered that increasing mitochondrial biogenesis via PGC-1α overexpression helped expedite recovery if applied post-injury [66]. Introducing increased mitochondria via PGC-1α overexpression prior to injury did not prevent injury or promote recovery, suggesting timing and quantity must be finely tuned [75].

Closely following this idea, another research group gathered transcriptional analysis from sepsis-associated AKI, which indicated oxidative phosphorylation genes were downregulated in addition to aberrant mitochondrial function [63]. More specifically, PGC-1α expression was reduced [63]. Global and tissue specific (proximal tubule) PGC-1α knockout mice lines were used in this study and results indicate normal kidney function until AKI where the animals then struggled to recover [63]. PGC-1α action seemingly acts downstream of TNFα induced inflammation as PGC-1α levels decreased after TNFα treatment, and PGC-1α rescued the oxygen consumption of TNFα treated nephron cells [63]. Another study found PGC-1α plays an important role in cellular recovery following an ischemia-reperfusion injury model [76]. Researchers treated with SRT1720 after AKI to agonize the SIRT1 pathway. Upon SIRT1 activation in rescued animals, the group found that PGC-1α had higher rates of deacetylation, an indicator of increased activation [76]. This study further substantiated the thought that PGC-1α could serve as a key therapeutic target for patients suffering AKI.

To this end, potential therapeutics were found in a study that identified trametinib (a MEK1/2 inhibitor) and erlotinib (an EGFR inhibitor) as potentially indirect PGC-1α modifiers that could help in the context of ischemia-reperfusion AKI [77]. In this study, treatment with trametinib blocked ERK1/2 and FOXO3a/1 phosphorylation and resulted in increased PGC-1α expression in kidney cells [77]. Additionally, linking EGFR to this pathway, authors found treatment with erlotinib also blocked ERK1/2 phosphorylation and exhibited a similar increase in PGC-1α expression as trametinib [77]. This study not only identified potential treatments of AKI, but also pinpointed a pathway including EGFR, ERK1/2, FOXO3a/1, and PGC-1α in the context of mitochondrial response to kidney injury.

Another very interesting relationship between a well-studied pathway and PGC-1α in the kidney has also been documented [66]. Here the researchers accumulated convincing evidence that the disease-causing gene *human nuclear factor 1B* (*HNF1B*) directly controls mitochondria via PGC-1α in kidney cells [66]. Using a similar sepsis-induced AKI model as previously discussed [65], this group found a comparable decrease in PGC-1α and a subsequent decline in other mitochondrial biogenesis genes as well as substantial decreased HNF1B expression and many of its targets [66]. Additional experiments found HNF1B deficiency caused reduced PGC-1α expression and was associated with corresponding mitochondrial morphological defects [66]. A chromatin immunoprecipitation assay found HNF1B binds to the PGC-1α promoter region in mouse kidney cells [66]. The authors believe this is conserved in human kidney cells as an HNF1B-mutant-patient sample had decreased PGC-1α expression [66]. This uniquely connects HNF1B to mitochondrial biogenesis via PGC-1α [66]. Results from this study importantly connect HNF1B to PGC-1α and merit additional research to explore the involvement of PGC-1α in HNF1B associated kidney diseases.

With previous publications finding potential upstream regulators of PGC-1α in response to AKI, a unique study identified the downstream factors of PGC-1α post-AKI [65]. Interestingly, they found post ischemic AKI PGC-1α-deficient mice present with decreased niacinamide (NAM) levels, fat accumulation, and are unable to regain baseline kidney function [65]. Through a number of elegant rescue studies, the authors found PGC-1α regulates renal recovery from AKI by controlling nicotinamide adenine dinucleotide (NAD) biosynthesis via NAM and that NAM was needed for B-hydroxybutyrate to increase prostaglandin E2 production [65]. The authors also found that this NAM relationship to AKI recovery was important during cisplatin induced AKI as well as ischemic induced AKI [65]. Additional research related to this topic discovered the loss of a mitochondrial-related gene-dynamin-related protein 1 (DRP1) benefited proximal tubule cells following ischemic AKI [78]. The benefits included renoprotection via activation of PGC-1α and the associated B-hydroxybutyrate pathway ultimately resulting in decreased inflammation [78]. Further supporting these links, another group found decreased PGC-1α expression in AKI context, specifically toxin-induced AKI [78]. Transcriptomic analysis pointed to PGC-1α acting upstream of many of the genes affected in folic acid induced AKI [79]. They found that decreased PGC-1α resulted in an increase in inflammation (including activation of NF-kB) and cell death [79].

### 3.3. Chronic Kidney Disease (CKD)

Chronic kidney disease (CDK) is the progressive decrease in renal function due to repeated insults over time [60]. In this section we will discuss both direct and indirect actions of PGC-1α in chronic kidney disease including diabetic nephropathy, glomerular function, and fibrosis.

As previously described, PGC-1α plays a significant role in type 2 diabetes mellitus in multiple tissues including muscle, pancreas, and the kidney. Most of what has been covered up to this point has focused on tissues other than kidney. Here, we will focus on the role of PGC-1α in diabetic nephropathy, also called diabetic kidney disease. Diabetic nephropathy is the leading cause of CKD and too often results in end stage renal disease [80,81]. The glomerulus is the first point of contact for function of the nephron as it filters the blood and passes the filtrate to the nephron tubule for modification. Typically, the function of the glomerulus is affected in diabetic nephropathy as patients exhibit lesions and adverse glomerular filtration rates [80]. Two important studies have indicated a role for PGC-1α and the resulting mitochondrial biogenesis being essential for proper kidney function in the case of diabetic kidney disease [82,83]. Sharma and colleagues (2013) utilized bioinformatics that directed them to discover decreased levels of PGC-1α in diabetic kidneys [82]. The other group also saw decreased mRNA levels of PGC-1α in diabetic kidneys [74]. Furthermore, stimulating PGC-1α expression in diabetic kidneys via AICAR treatment rescued superoxide production in kidneys in addition to a number of clinical outputs [83].

With the evidence supporting an essential role for PGC-1α in the nephron to protect against diabetic nephropathy it is important to understand the possible molecular components regulating the expression of this factor. Upon completing RNA sequencing on glomeruli from a diabetic mouse model, a research team found the long noncoding RNA taurine-upregulated gene 1 (Tug1) was differentially expressed [84]. Additional experiments found Tug1 interacts with PGC-1α to promote PGC-1α expression and resulted in a rescue of downstream PGC-1α targets [84]. Though, another research group found the levels of PGC-1α have to be fine-tuned to allow a beneficial and not a deleterious outcome [85]. There exists a unique balance of PGC-1α expression required for basal glomerulus function [85]. Both mouse and humans suffering from diabetic kidney disease had decreased expression of PGC-1α and the corresponding mitochondrial transcripts, most likely caused by the inhibitory action of TGF-B [85]. Using an inducible nephron-specific PGC-1α overexpression line, the authors found excess PGC-1α causes collapsing glomerulopathy including albuminuria and renal failure/glomerulosclerosis [85]. This suggests a delicate balance of PGC-1α is necessary for proper nephron function.

While these studies strongly indicate a vital role of PGC-1α another group used an inducible nephron specific PGC-1α knockout mouse model to study the physiological role of PGC-1α in the kidney [86]. Results indicated the role of PGC-1α in mitochondrial metabolic systems is observed in the kidney similar to the previously described roles of PGC-1α in other tissues. After inducing the knockout at 12 weeks, researchers noted abnormal sodium excretion, most likely due to the decreased protein expression of sodium transporters NCCT and NKCC2 [86]. These phenotypes ultimately led to mice presenting with renal steatosis, although the authors hypothesize PGC-1α is dispensable for renal physiology due to mild phenotypes and high survival rates [86].

In chronic kidney disease, there exists progressive fibrosis, which leads to the decreased ability of the kidney to perform its tasks as previously functional tissue is converted to nonfunctional connective tissue. Various studies have found PGC-1α plays a direct role in the fibrotic response to repeated insults to the kidney. Renal fibrosis leading to CKD and eventually end stage renal disease can largely be attributed to mis-regulated expression of inflammation and metabolism pathways according to transcriptomics work presented by Kang et al. (2015) [87]. Specifically, they observed fatty acid oxidation components (including PGC-1α specifically) were decreased in the case of fibrosis [87]. This ultimately results in three characteristics of fibrosis: decreased ATP, cell death, and dedifferentiation [87]. Furthermore, they observed molecular connections including evidence that TGFB1 reduced PGC-1α expression via SMAD3 [87]. Interestingly, PGC-1α overexpression was able to rescue a many fibrotic phenotypes leading the field to further pursue a possible targeted treatment for fibrosis progression [87]. This genetic relationship was further supported by a study that also found PGC-1α acts downstream of TGF-B [88]. Once again, there was an adaptive response using PGC-1α in the reaction to stress [88]. Researchers showed that exercise was a possible indicator of more positive outcomes in CKD patients [88]. The mode of action uncovered in this study involves the myokine irisin, which acts to inhibit TGF-B type 1 receptor allowing PGC-1α to decrease the amount of damage to kidneys [88]. PGC-1α expression improves metabolic function and decreases fibrosis in mice kidneys [88].

Understanding renal fibrosis is a key step to formulate innovative therapeutics to combat CKD. Another research team found fatty acid oxidation genes, including PGC-1α were downregulated in fibrotic kidneys due to Notch signaling. In fact, PGC-1α expression was decreased in several types of kidney disease including toxin-induced AKI, physical obstruction induced kidney injury, and a genetic CKD model. The authors found evidence suggesting that the Notch component Hes1 represses PGC-1α in kidney cells. Additionally, PGC-1α expression was able to rescue a number of ailments including kidney physiology, fibrotic gene expression, mitochondrial physiology, and fatty acid oxidation both in vivo and in vitro [67]. A year later, the same group then found a unique role for PGC-1α downstream factor mitochondrial transcription factor A (Tfam) in kidney fibrosis [89]. Using a combination of transcriptomics, loss of function studies, and rescue experiments, the researchers used a number of kidney disease models to support Notch signaling components Jag1 and Notch2 are key players in fibrosis by negatively affecting Tfam, ultimately resulting in aberrant metabolic function [89].

### 3.4. Future Direction: Polycystic Kidney Disease (PKD) and Cilia

One intriguing area of future studies could be focused on the relationship between PGC-1α and ciliogenesis. There is overlap between kidney diseases and ciliopathies including conditions such as polycystic kidney disease (PKD) and nephronophthisis. Although significant progress has been made in understanding disease progression, the molecular mechanisms and intertwining pathways involved in cilia formation and function is an area of extreme importance as PKD affects over 12 million people worldwide [90]. PKD presents with many fluid-filled sacs (cysts) that develop in the kidneys rendering it more difficult to properly filter the blood and produce urine. PKD is a genetic disorder that affects around 500,000 people in the United States alone [90]. Although two main causative ciliary genes have been identified, there is no cure for PKD and the molecular consequences involving cilia are not well understood. The genetic mechanisms that are known focus on mutations in the genetic drivers polycystin-1 (PKD1) and polycystin-2 (PKD2) that result in cyst formation once a certain threshold of insufficient protein product is reached [91,92,93,94,95]. Both polycystin proteins localize to the ciliary body and function in chemosensing by regulating calcium levels. While this information is important, the current gap in knowledge that needs to be addressed is identifying other genes these polycystin proteins interact with as well as the modes of interaction, either direct or indirect [93,94,95].

The likely association between PKD and PGC-1α has been supported by researchers who noted that oxidative stress is present early in PKD and mitochondria is involved in pathogenesis of this disease. They discovered a quantitative decrease in PGC-1α mRNA and protein expression that was supported further by histological staining of cyst-lining cells [96]. Additional experiments indicate this PGC-1α dependent mitochondrial dysfunction was caused by decreased calcium via calcineurin/p38-MAPK signaling [96]. The authors were able to rescue superoxide production by supplementing PKD1 mutant cells with MitoQuinone, a mitochondrion antioxidant [96]. The study later proposes that PGC-1α could play a pivotal role in PKD by recapitulating the molecular function of MitoQuinone, where it controls mitochondrial superoxide production and oxidative stress [96]. An interesting link between PGC-1α, its well-studied molecular partners, and PKD has been reported [97]. Here, the authors found evidence that microRNA17 promotes cyst progression in PKD by inhibiting mitochondrial metabolism [97]. In this study, they observe decreased PGC-1α expression in both PKD1 and PKD2 knockout mice [97]. They go on to propose upregulation of c-myc by PKD1/2 results in enhanced activity of miRNA-17, which then functions to inhibit mitochondrial metabolism via the PGC-1α partner PPAR α [97].

While studies of PGC-1α knockouts in mice do not result in cystogenesis, there are a number of reasons to continue investigations. Some of these reasons include: the possibility of PGC-1β compensation [41], the unending connections of PGC-1α pathway components and their relationship to PKD etiology and progression [98] and compelling data from recent studies using zebrafish pronephros where loss of PGC-1α induces cystogenesis in the kidney [99]. Also, PGC-1α has been linked to several PKD/cilia related genes including Hnf1b [66,100,101], Notch signaling [67], prostaglandin signaling [65,99,102,103], and mTOR signaling [26,104]. Specifically, there is mounting evidence mitochondria and the resulting metabolic effects need to strike the balance seen in a number of examples already mentioned in order to properly form cilia [105,106]. The connections between PGC-1α, mitochondria, cilia, and diseases such as PKD and nephronophthisis is an area of research primed to shed light on underlying genetic and cellular mechanisms contributing to disease states.

## 4. Conclusions

Members of the PGC-1 family are known to be expressed in tissues with oxidative metabolism demands and also dynamically expressed in response to physiological stimuli. PGC-1 coactivators serve diverse functions because they can interact with many binding partners. Among the PGC-1 family members, PGC-1α has incredible versatility to affect cellular processes by virtue of its many transcription factor targets. This versatility also has a role in a multitude of disease contexts throughout the body, including in the liver, nervous system, and kidneys, among others. Here, we have discussed the roles of PGC-1α in a number of contexts including its role as an adaptive response to energy requirements, the current understanding of PGC-1α in disease contexts, with a special focus on the kidneys, and future areas of research relating PGC-1α and mitochondrial deficiencies to PKD. There exists a large challenge when studying PGC-1α knockout models, as recent findings suggest tissue-specific isoforms that may not be detectable by certain analyses (e.g., PCR amplifying or using antibodies for areas of the PGC-1α sequence that is not specific to the tissue of interest) [52]. This variety of transcript variants could result in compensation in knockout murine models, which have historically targeted exons 3-5 [12,46,63], although there are a number of PGC-1α transcript variants that do not include these exons. A fantastic example of this temporal spatial specificity of PGC-1α can be observed in a study that found conditional loss of PGC-1α in adult mice results in decreased dopaminergic neurons in particular regions of the brain [107].

The dynamic expression and roles of PGC-1α and the associated transcript variants give reason to question if PGC-1α may be involved in an even greater number of processes in mammalian physiology. Interestingly, another possibility exists that PGC-1β may compensate for loss of mammalian PGC-1α [41]. It does not appear that this compensation exists in all vertebrates as PGC-1α deficient zebrafish exhibit more severe phenotypes than their mammalian counterparts [61,99]. These differences do not mean there are not also strong similarities as some very specific phenotypes are observed in both mammalian and zebrafish nephrons. Specifically, loss of PGC-1α results in a decrease of expression in the transporter Slc12a3 (NCCT) in zebrafish and mice [61,86]. With the evidence of genetic compensation in Lai et al., (2008) and identification of multiple tissue-specific isoforms [41,51,52,54], future research could seek to identify additional isoforms and ensure complete knockout while also accounting for potential compensatory action of PGC-1β.

## Abbreviations

AKI (acute kidney injury), ALS (amyotrophic lateral sclerosis), AMPK (5’ adenosine monophosphate-activated protein kinase), ATF2 (Activating Transcription Factor 2), BHD (Birt-Hogg-Dube syndrome), CaMKIV (calcium/calmodulin-dependent protein kinase IV), CaN (calcineurin A), ccRCC (clear cell renal carcinoma), CKD (chronic kidney disease), CREB (cAMP response element-binding protein), DRP1 (dynamin-related protein 1), EGFR (epidermal growth factor receptor), EMT (epithelial-to-mesenchymal transition), ER (estrogen receptor), ERK1/2 (Extracellular Signal-Regulated Kinase 1/2), ERR (estrogen related receptor), FLCN (folliculin), FOXO3a/1 (Forkhead Box O3), glucocorticoid receptor (GR), GLUT4 (glucose transporter 4), HAT (histone acetyltransferase), HNF1B (human nuclear factor 1B), HNF4α (hepatocyte nuclear factor-4 alpha), MEF2C/2D (myocyte enhancer factor 2C and 2D), MEK1/2 (Dual specificity mitogen activated protein kinase kinase 1), MLXIPL (MLX Interacting Protein Like), MYBBP1A (MYB Binding Protein 1a), NAD (nicotinamide adenine dinucleotide), NAM (niacinamide), NCCT or Slc12a3 (sodium-chloride symporter), NF-kB (nuclear factor kappa-light-chain-enhancer of activated B cells), NKCC2 (sodium potassium chloride cotransporter), NRF-1 (Nuclear Respiratory Factor 1), NRF-2 (Nuclear Respiratory Factor 2), OXPHOS (Oxidative phosphorylation), p38 (p38 mitogen-activated protein kinase), PGC-1α (Peroxisome proliferator-activated receptor gamma co-activator 1 alpha), PKD (polycystic kidney disease), PKD1 (polycystin-1), PKD2 (polycystin-2), PPARγ (peroxisome proliferator-activated receptor gamma), PPARα (peroxisome proliferator-activated receptor alpha), PRC (PGC-related coactivator), RG (glucocorticoid receptor), ROS (reactive oxygen species), RXRα (retinoid X receptor alpha), SIRT1 (Sirtuin 1), SMAD3 (Mothers against decapentaplegic homolog 3), SOD2 (Superoxide dismutase 2), TFAM (mitochondrial transcription factor A), TGF-B (Transforming growth factor beta), THR (thyroid hormone receptor), TNFα (Tumor necrosis factor alpha), Tug1 (taurine-upregulated gene 1), UCP1 (uncoupling protein-1), VEGF (Vascular endothelial growth factor), Vhl (Von Hippel-Lindau)
